# Cortical Hand Bone Porosity and Its Association with Distal Radius Fracture in Middle Aged and Elderly Women

**DOI:** 10.1371/journal.pone.0068405

**Published:** 2013-07-03

**Authors:** Alvilde Dhainaut, Mari Hoff, Unni Syversen, Glenn Haugeberg

**Affiliations:** 1 Department of Neuroscience, Division of Rheumatology, Norwegian University of Science and Technology, Trondheim, Norway; 2 Department of Rheumatology, St. Olav’s Hospital, Trondheim, Norway; 3 Department of Cancer Research and Molecular Medicine, Norwegian University of Science and Technology, Trondheim, Norway; 4 Department of Endocrinology, St. Olav’s Hospital, Trondheim, Norway; 5 Department of Rheumatology, Hospital of Southern Norway, Norway; University of Southampton, United Kingdom

## Abstract

**Objective:**

Reduced bone mineral density (BMD), assessed by Dual Energy X-ray absorptiometry (DXA), is a well-known risk factor for fragility fracture. A large proportion of patients with fracture have only slightly reduced BMD. Assessment of other bone structure features than BMD may improve identification of individuals at increased fracture risk. Digital X-ray radiogrammetry (DXR), which is a feasible tool for measurement of metacarpal cortical bone density, also gives an estimate of cortical bone porosity. Our primary aim was to explore the association between cortical porosity in the hand assessed by DXR and distal radius fracture.

**Methods:**

This case-control study included 123 women >50 years with distal radius fracture, and 170 controls. DXR was used to measure metacarpal BMD (DXR-BMD), cortical porosity (DXR-porosity), thickness (DXR-CT) and bone width (DXR-W) of the hand. Femoral neck BMD was measured by DXA.

**Results:**

The fracture group had a statistically significant lower DXR-BMD (0.492 vs. 0.524 g/cm^2^ p<0.001), higher cortical DXR-porosity (0.01256 vs. 0.01093, p<0.001), less DXR-CT (0.148 vs. 0.161cm, p<0.001) and lower femoral neck DXA-BMD (0.789 vs. 0.844 g/cm^2^, p = 0.001) than the controls. In logistic regression analysis adjusted for age, a significant association with distal radius fracture (OR, 95% CI) was found for body mass index (0.930, 0.880–0.983), DXA-BMD (0.996, 0.995–0.999), DXR-BMD (0.990, 0.985–0.998), DXR-porosity (1.468, 1.278–1.687) and DXR-CT (0.997, 0.996–0.999). In an adjusted model, DXR-porosity remained the only variable associated with distal radius fracture (1.415, 1.194–1.677).

**Conclusion:**

DXR derived porosity is associated with fracture at distal radius and might be a sensitive marker for skeletal fragility.

## Introduction

Distal radius is one of the most common sites for osteoporotic fractures in middle aged and elderly women [1]. Low bone mineral density (BMD) has been identified as a major and independent risk factor for distal radius fracture [2]. However, a large proportion of fragility fractures occur in women with slightly reduced BMD and even in women with normal BMD [3], [4]. Other factors also contribute to bone strength (e.g. bone geometry, bone porosity and bone material properties) [5]–[8]. Due to technological improvement, our understanding of fracture risk at sites dominated by cortical bone has developed beyond consideration of bone density alone [9]–[13].The digital X-ray radiogrammetry (DXR), a computer version of the traditional metacarpal radiogrammetry, is a feasible method developed primarily for the estimation of metacarpal cortical hand BMD on hand radiographs [14]. DXR-BMD has been shown to correlate with Dual Energy X-ray Absorptiometry (DXA) BMD at femoral neck [15] and to be a reliable predictor of distal radius fracture [16]. The DXR method also gives an estimate of cortical bone porosity. The main objective of this study was to explore the association between increased cortical bone porosity assessed by the DXR software (DXR-porosity), and distal radius fragility fracture in middle-aged and elderly women.

## Materials and Methods

### Study Ethics

The study was approved by the Regional Committee for Medical Research Ethics South-East Norway (previous REK Sørlandet now REK Sør-Øst Ref: S-03207) and the Norwegian Data Inspectorate (Datatilsynet Ref: 200300837).

All participants provided a written informed consent to participate in the study.

### Subjects

In this case-control study, women (>50 years) with a recent fragility fracture at the distal radius were compared with controls from the general population. The distal radius fracture patients were consecutively recruited from a community hospital located in Southern Norway in the two year period from 1^st^ of January 2004 to 31^st^ of December 2005. The patients were participating in a clinical study on fracture in middle-aged and elderly women [17], [18]. In this study, a low-energy fracture was defined as resulting from minimal trauma (e.g. falling from a standing height or less). The fracture at distal radius should be located within 3 cm from the radio carpal joint.

In the two-year period, a total of 278 women were identified with a fragility fracture at the distal radius. These women were invited for osteoporosis and fracture risk assessment at the hospital’s Osteoporosis center, a fracture liaison center. From the 278 identified patients, 218 were assessed at the Osteoporosis center. Those who were not assessed were deemed unable to attend by health care personnel, on grounds of severely impaired physical or mental health, being tourists or had chosen to decline the invitation. The 60 patients not assessed for osteoporosis were in mean eight years older than the group of 218 women assessed for osteoporosis (75.6 vs.67.2 years, p = <0.001).The median time from fracture to assessment at the Osteoporosis center was ten days (inter-quartile range eleven days).

A total of 321 female controls were randomly identified in the National registry for the same catchment area and invited to attend by mail. The controls were intended to be matched for age. Of these, 191 accepted and were assessed at the Osteoporosis center. The matching process was not complete, especially for the oldest patients. We used the whole control group, thus patients were not pair matched. At the Osteoporosis center, demographic and clinical data were collected for fracture risk assessment. The data were obtained partly by self-reported questionnaires and partly by interview and clinical examination performed by trained nurses. For controls with lacking information about previous fracture, from questionnaires, the medical records at the hospital were reviewed. The hospital is the only center treating fracture patients in the geographic area. DXA BMD was measured at the Osteoporosis center. Radiographs for DXR assessment of the fracture patients were taken at the emergency department simultaneously when radiographs for diagnosing fracture were performed. For the controls the hand radiographs for DXR assessment were taken at inclusion.

Among the 409 women assessed, 308 had radiographs available for DXR assessment. There were no significant differences between women who had and those who did not have radiographs available for age (67.4 vs. 69.1 years, p = 0.15), height (164.5 vs. 163.9 cm, p = 0.38) and weight (70.6 vs. 68.6 kg, p = 0.15). For 15 women, radiographs could not be analyzed because of bad positioning or hand deformities. The final study population consisted of 123 women with a recent distal radius fracture and 170 women recruited from the population. All were Caucasians.

### Bone Density Measures

The hand radiographs for DXR assessment were taken with a Fuji FCR XG1 (CR; FFD 100cm; tube voltage 50 kV; exposure dose 5 mA) which give a picture resolution of 0.100 mm/pixel. The non-dominant hand was assessed when possible. Of the 293 women, 269 had BMD assessed. Cortical hand bone measures were assessed by dxr-online (Sectra, Linköping, Sweden). The dxr-online applies the same image analysis algorithms as the Pronosco X-posure System, previously described by Rosholm et al [14]. The computer software recognizes regions of interest (ROI) around the narrowest part of the 2^nd^, 3^rd^ and 4^th^ metacarpal bones on digital hand X-rays. Within each ROI, the endosteal (inner) and periosteal (outer) edges are identified, and then the average cortical thickness (DXR-CT) and bone width (DXR-W) are determined for each metacarpal bone. A bone volume per projected area (VPA) is computed for each of the three metacarpal bones assuming they are cylindrically shaped bones. The cortical porosity measure for the DXR method is based on a digital estimation of the fraction of the cortical bone volume that is not occupied by bone. Local intensity minima (“holes”) in the cortical bone regions are found using a recursive (climbing) algorithm, starting from the outer region. A combined porosity measure is derived from the area percentage of holes found in the cortical part relative to the entire cortical area, by averaging over the involved bones and scaled to reflect a volumetric ratio rather than the projected area. The formula for calculating DXR-BMD is as follows: c*VPA*(1-P) where c is a scaling constant, VPA is a weighted average of the bone volume per projected area of each metacarpal and P is the combined porosity measure, the DXR-porosity. The scaling constant c is determined so that DXR-BMD is, on average, equal to that of the mid-distal forearm region of the Hologic QDR 2000 densitometer (Hologic, Waltham, MA, USA) [14].

BMD was measured at the femoral neck by DXA, using Lunar Prodigy with enCORE software (GE Healthcare, Madison, WI, USA). Osteoporosis was defined as a T score≤−2.5 at femoral neck [19], [20].

Trained nurses performed all the bone density measurements using standardized protocols.

The precision, expressed as the coefficient of variation (CV), based on repeated measurements of cadaver hand phantoms, was 2.94% for DXR-porosity and 0.22% for DXR-BMD, whereas the in-vivo CV was 0.46% for DXR-BMD. For DXA the CV for long-term spine phantom measurements was 0.62% and for short-term in-vivo femoral neck, the CV was 1.56%.

### Statistical Analysis

Continuous variables were presented as mean with standard deviation and categorical variables as numbers. Normality was checked by visual inspection of qq plot. For group comparison, we used t-test for continuous variables and chi-square test for categorical variables.

To explore the association between DXR-porosity and fragility fracture as a dependent variable, we used logistic regression analysis. We adjusted for possible confounders which may have affected both porosity and fracture risk (e.g. clinical factors such as age, body mass index (BMI), chronic disease, and medication). We also adjusted for BMD (DXA-BMD and DXR-BMD). The other sub factors from the DXR-BMD algorithm, cortical thickness (DXR-CT) and bone width (DXR-W) were also tested. We first performed logistic regression analyses for all tested variables adjusting for age. We then analysed DXR-porosity, adjusted for each of the variables, tested as pairs one-by-one, and also adjusted for age. Finally, DXR-porosity was tested in a multiple regression model. In the logistic regression analysis, we used as one unit change: mg/cm^2^ for the BMD measurements, a thousandth for DXR-porosity, and µm for DXR-CT and DXR-W, to give a more clinically meaningful interpretation of the data. Due to collinearity, the DXR measures; porosity, cortical thickness and bone width used to calculate DXR-BMD, were explored in separate logistic regression analyses for their association with distal radius fracture. Receiver operating curves (ROC) analyses were performed to examine for differences in the ability of DXR-BMD, DXR-porosity and DXR-CT to separate between distal radius fracture participants and controls. We also explored for a relationship between cortical DXR-porosity, number of fractures in the entire population of fracture patients and controls, and the BMD categories normal, osteopenia and osteoporosis as defined by DXA of the femoral neck.

Statistical tests were performed using PASW Statistics 18 (IBM SPSS statistics) except for the ROC analysis. For these analyses, testing differences in area under the curve (AUC) between DXR bone variables, we used the free Star software (http://melolab.org/star/home.php) [21]. Significance level was p<0.05.

## Results

Demographic, clinical and bone measure data for the control and the distal radius fracture group are shown in Table 1. None of the women with missing data from the self-reported questionnaire on history of previous fracture had a fracture reported in their medical records. A statistically significant difference between the two groups was found for weight, BMI and DXA femoral neck BMD, as well as DXR-BMD, DXR-porosity, and DXR-CT in the hand, but not for DXR-W.

**Table 1:** Characteristics of the women with distal radius fracture and the control group.

	FractureN = 123	ControlsN = 170	P
Age, years	68.0 (10.1)	67.1 (8.7)	0.431
Height, cm	164.9 (5.8)	164.2 (6.4)	0.337
Weight, kg	68.7 (12.9)	72.1 (13.7)	0.031
BMI, kg/m^2^	25.3 (4.5)	26.7(4.5)	0.008
Smoking	16/120*	17/169*	0.454
Rheumatoid arthritis	3/123	1/170	0.313
Chronic disease a	21/123	26/170	0.748
Menopause <45 years	16/108*	14/161*	0.118
Previous fracture	40/120*	50/164*	0.943
Parent fracture	53/118*	70/169*	0.628
History of falls	49/108*	58/140*	0.534
Exercise b	92/119*	123/169*	0.412
Excessive alcohol	0/123	0/170	–
Glucocorticoids	10/123	9/169*	0.337
Osteoporosis treatment c	37/120*	51/170*	0.916
DXA BMD, g/cm^2^	0.789 (0.123)	0.844 (0.136)	0.001
DXR BMD, g/cm^2^	0.492 (0.074)	0.524 (0.075)	<0.001
DXR porosity	0.01256 (0.00023)	0.01093 (0.00021)	<0.001
DXR CT, cm	0.148 (0.028)	0.161 (0.031)	<0.001
DXR W, cm	0.823 (0.047)	0.812 (0.028)	0.067

Characteristics of the women with distal radius fracture and the control group: Continuous variables are presented as mean with standard deviation and categorical variables as numbers.

* The denominator differs from 123 in the fracture group and 170 in the control group due to missing data.

a Inflammatory or endocrine disease (fracture group vs. control group): other rheumatic diseases than rheumatoid arthritis (2 vs. 2); asthma/chronic obstructive lung disease (10 vs. 9); diabetes mellitus (4 vs. 8); hyperparathyroidism (1vs.1); hypothyroidism (5 vs. 9); chronic kidney disease (1vs.0).

b Exercise more than 30 minutes three times a week.

c Calcium/vitamin D (29 vs. 17); bisphosphonates, selective oestrogen receptor modulator, or oestrogen (22 vs. 16).

BMI: body mass index; DXA: dual energy X-ray absorptiometry; BMD: bone mineral density; DXR: digital X-ray radiogrammetry; CT: cortical thickness; W: bone width.

In Table 2 the association between cortical DXR porosity and current distal radius fracture is displayed. The simple regression analysis adjusted for age showed a statistically significant association with distal radius fracture for BMI, DXA femoral neck BMD, DXR-BMD, DXR-porosity, and DXR-CT. No association was found for DXR-W.

**Table 2:** Association between cortical DXR-porosity and current distal radius fracture.

	Simple regression^a^		DXR porosity and possible confounders one by one^a^		Multiple regression^a^	
Variables	OR (95% CI)	P	AdjustedOR (95% CI)	P	Adjusted OR (95% CI)	P
DXR porosity	1.468 (1.278−1.687)	<0.001	−	−	1.415 (1.194−1.677)	<0.001
DXR porosity BMI (kg/m2)	***−***0.930 (0.880−0.983)	0.010	1.452 (1.262−1.672) 0.947 (0.894−1.003)	<0.0010.065	***−***0.945 (0.879−1.017)	0.129
DXR porosity Smoking	1.408(0.678−2.925)	0.358	1.468(1.285−1.703) 1.300(0.723−2.335)	<0.001 0.381	1.133(0.486−2.643)	0,772
DXR porosity RA	***−***4.133 (0.424−40.28)	0.222	1.473 (1.281−1.693) 4.856 (0.467−50.488)	<0.0010.186	***−***4.460 (0.367−54.26)	0.241
DXR porosity Chronic diseases^b^	−1.097 (0.580−2.074)	0.775	1.467 (1.276−1.685) 1.388 (0.213−2.789)	<0.0010.348	−1.896 (0.842−4.270)	0.123
DXR porosity Menopause <45 years	***−***1.824 (0.850−3.916)	0.123	1.422 (1.233−1.641) 1.443 (0.644−3.236)	<0.0010.373	***−***1.423 (0.581−3.485)	0.440
DXR porosity Exercise^c^	−1.296 (0.447−2.251)	0.356	1.479 (1.285−1.703) 1.300 (0.723−2.335)	<0.0010.381	−1.462 (0.735−2.907)	0.279
DXR porosity GC current	−2.948 (0.719−12.08)	0.133	1.475 (1.276−1.704) 4.480 (0.865−23.216)	<0.0010.074	−2.881 (0.473−17.550)	0.251
DXR porosity OPO treat n/y^d^	−1.012 (0.608−1.685)	0.963	1.474 (1.282−1.695) 1.086 (0.629−1.877)	<0.0010.767	***−***1.181 (0.633−2.2074)	0.601
DXR porosity DXA BMD femoral neck mg/cm2	−0.996 (0.995−0.999)	0.001	1.433 (1.230−1.669)^a^ 0.999(0.996−1.001)	<0.0010.350	−0.999 (0.996−1.002)	0.587
DXR porosity DXR BMD mg/cm2^e^	−0.990 (0.985−0.998)	0.001	1.621 (1.288−2.041)^a^ 1.005 (0.996−1.013)	<0.0010.280		
DXR porosity DXR CTµm e	−0.997 (0.996−0.999)	<0.001	1.565 (1.240−1.976)^a^ 1.001 (0.999−1.003)	<0.0010.495		
DXR porosity DXR W µm^e^	−1.000 (1.000−1.001)	0.084	1.455 (1.266−1.672)^a^ 1.000 (1.000−1.001)	<0.0010.256		

Association between cortical DXR-porosity and current distal radius fracture tested in unadjusted and adjusted logistic regression models.

a Adjusted for age.

b Inflammatory or endocrine disease (fracture group vs. control group): other rheumatic diseases than rheumatoid arthritis (2 vs. 2); asthma/chronic obstructive lung disease asthma (10 vs. 9); diabetes mellitus (4 vs. 8); hyperparathyroidism (1 vs. 1); hypothyroidism (5 vs. 9); kidney disease (1 vs. 0).

c Exercise more than 30 minutes three times a week.

d Calcium/vitamin D (29 vs.17); bisphosphonates, selective oestrogen receptor modulator, or oestrogen (22 vs.16).

e Due to collinearity between the DXR measures porosity, BMD, CT and W these variables are not in the presented multivariable analysis.

DXR: digital X-ray radiogrammetry; BMI: body mass index; RA: rheumatoid arthritis; GC: glucocorticoid; DXA: dual energy X-ray absorptiometry; BMD: bone mineral density; CT: cortical thickness; W: bone width.

When DXR-porosity was adjusted for age and for each of the variables listed in Table 2 one by one, the association between DXR-porosity and distal radius fracture remained statistically significant (Table 2). When each disease in the chronic disease variable was tested separately, the same result for DXR-porosity was found (data not shown).

In the final multivariable model as shown in table 2, DXR- porosity was the only variable that remained significantly associated with the presence of distal radius fracture apart from age (data not shown). When DXA-BMD, DXR-BMD or DXR-CT were tested as the sole bone measure variable, in multivariable analysis excluding DXR-porosity, each of them was associated with distal radius fracture (data not shown). In the multivariable analysis presented, we excluded DXR-BMD, DXR-CT and DXR-W due to collinearity with DXR-porosity. When applying ROC analysis to examine the ability to distinguish between distal radius fracture patients and controls, the AUC for DXR-porosity (0.715) was statistically significantly higher than the AUC for both DXR-BMD (0.652, p = 0.003) and DXR-CT (0.660, p = 0.007). AUC for DXA-femoral neck was 0.616.

**Figure 1:**
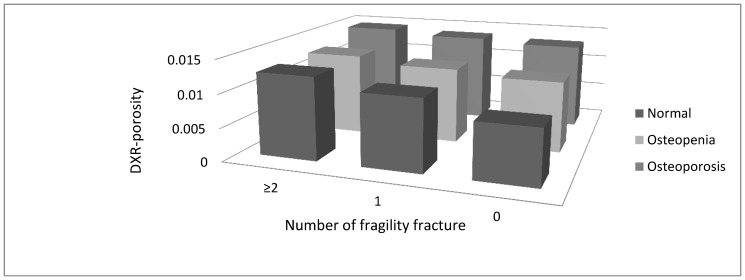
Relationship between cortical digital X-ray radiogrammetry porosity, number of fragility fractures and the categories of normal, osteopenia and osteoporosis as defined by femoral neck dual energy X-ray bone density.

As noted in Table 1, 30% of the women in the control group and 33% in the fracture group reported having had a previous fracture. Previous fracture included any kind of fragility fracture after 50 years of age. Combining the two groups, 128 women were found to have had one fracture, 35 had two, nine had three and one had four fractures. A total of 120 women in the control group had never experienced a fracture. A statistically significant difference in DXR-porosity was seen between women who had never experienced a fracture and those with one fracture (0.010368 vs. 0.012054, p<0.001), and between those with one fracture and ≥2 fractures (0.012054 vs. 0.013678, p<0.001). When all women were analyzed as one group and divided according to normal, osteopenic or osteoporotic status at the femoral neck, we observed the same tendency of higher DXR-porosity being related to increasing numbers of fractures (Figure 1). This was most pronounced for individuals with normal femoral neck DXA-BMD. For women with osteoporosis and osteopenia, there were no statistically significant differences in DXR-porosity between those with no fracture history and those with one fracture, nor between women with one and two or more fractures. However, for women with normal femoral neck BMD, a significant difference in DXR-porosity was found between those with no fracture (n = 40) and those with one fracture (n = 22) (0.008300 vs. 0.0108408, p<0.001), and between no fracture and two or more fractures (n = 3) (0.008300 vs. 0.012511, p = 0.003). There was also a difference in DXR-porosity between women with one fracture and women with two or more fractures (0.010841 vs. 0.012511). This difference, however, did not reach statistical significance (p = 0.10), due to the low number of women with two or more fractures in the group with normal femoral neck BMD (n = 3).Similar results were seen when the six women in the control group with no history of previous fracture based on information from medical records were withdrawn from the analysis.

## Discussion

The main finding from this case-control study was the demonstration of a significant and strong association between increased porosity at metacarpal cortical bones assessed by DXR and distal radius fracture in women.

Although the DXR-porosity measure is a less sensitive measure, it consistently distinguished between women with current distal radius fracture and controls, including when adjusted for other possible confounders such as age, BMI, smoking and medication. Further, it remained significant when adjusted for BMD assessed by DXA and DXR-BMD. Our finding that cortical porosity was associated with fracture is in agreement with previous studies using more sensitive measures for porosity such as micrographs and HRpQCT. In the literature, trabecular and cortical architecture has been reported to be impaired in postmenopausal women with fractures [9] and cortical porosity has been found to increase with age [10], [11]. In studies using HRpQCT a variation in cortical porosity in subjects with identical BMD has been found [12], and older persons have been found to have increased cortical porosity compared to younger persons with similar BMD [13].

In a recent publication by Patsch et al, diabetic women with fragility fractures were found to have an increased cortical porosity assessed by HRpQCT compared to diabetic women without fracture [22]. In this study they also found a non-significant increase in non-diabetic women with fracture compared to women without fracture. The lack of significance in this group may have been due to the low number (n = 20 in each group) of non-diabetic patients examined [22]. In our study there were a small number of women with diabetes; four distal radius fracture participants and eight controls. These were included in the collective parameter “chronic disease”. We also performed the calculation with adjusting for diabetes as a single variable and DXR-porosity remained significantly associated to fracture.

Interestingly, in a previous small study of postmenopausal women using a former version of DXR, significant differences were found for both DXR-BMD and DXR-porosity between women treated and not treated with bisphosphonates, whereas no significant change was seen for DXA-BMD [23]. This indicates that cortical DXR-porosity can be modified and thus be responsive to treatment.

Norway has been shown to have the highest incidence of distal radius fracture worldwide [24], [25]. Differences in distal radius fracture incidence between the ethnic Norwegian and the Asian immigrant population have been reported from Oslo [25]. Asian women have been reported to have lower fracture incidence despite their lower BMD [26]. An interesting question is if these differences can be explained by other bone properties such as porosity, or if they are related to other factors, such as living habits or genetic differences. In a study from USA bone microarchitecture in postmenopausal women showed differences in cortical thickness between ethnic Chinese and ethnic white women. Chinese women had smaller bone size and thicker bone cortex [26], [27]. Thus, future fragility fracture studies in ethnic groups should explore other bone properties than BMD to increase our understanding and ability to identify patients at increased fracture risk. This is further highlighted by the fact that most patients with fragility fracture at the distal radius have osteopenia and may even have normal BMD according to the WHO criteria [2]. In a Norwegian multicentre study of 1576 women with distal radius fragility fracture, 34% had osteoporosis, 50% had osteopenia and 16% had normal BMD, according to the WHO criteria for osteoporosis based on DXA at femoral neck [4].

In our study on Norwegian women with Caucasian ethnicity, we found that the difference in cortical porosity between those with and without fracture seems to be more important in women with a normal DXA-BMD than in women with osteoporosis as shown in Figure 1. This may indicate that especially in patients with osteopenia and normal DXA-BMD, cortical porosity, as measured by DXR, may help to improve identification of women at high risk of fracture.

A major advantage for the DXR method is that it only requires a hand X-ray, widely available and with a low radiation dose of 3.7 microSV [28]. Furthermore, the X-rays can be taken at the same time as fracture evaluation.

Strengths of our study include that the wrist fracture participants come from a rather broad spectrum of individuals with distal radius fracture. Participants were recruited from an osteoporosis fracture liaison outpatient clinic, and were therefore representative of the distal radius fracture population. Furthermore, the data collection and BMD measurements were performed soon after fractures (within a median time of ten days).

Our study does have limitations. This is a case-control study and ideally a prospective and longitudinal study design may have been a better method. However, such a study design would have required far more resources. Despite that patients and controls were recruited prospectively into the study a total of 101 women did not have hand radiographs available for measurements of DXR- porosity. This was explained by the two following main reasons: First a large number of patients did not have a hand radiograph performed when assessed at the emergency unit and second some radiographs were not able to be retrieved when hand radiographs were reanalysed for DXR- porosity, this due to change in the hospital archive system. The DXR-porosity measure that was used also has limitations. As described in the method section, the DXR-porosity measure assumed to reflect the pores in the cortical bone is based on a digital estimation of the fraction of the cortical bone volume that is not occupied by bone. With a conservative scaling, this measure is typically <2% [14]. With more sensitive measures of bone structure, the proportion of pores in cortical bone has been reported to be 5–14% of the cortical bone volume [13], [29], [30]. This clearly illustrates that the DXR-porosity measure has limited resolution and only provides a rough measure because it only detects large pores in the cortical bone compared with methods with higher resolution (e.g. HRpQCT). In a previous study we showed that the DXR-BMD differs between modalities and that the modalities with better resolution measure a lower DXR-BMD. This may be explained by a higher measured porosity [31]. Ideally we should have used an X-ray modality with a better resolution but the X-rays for DXR assessment were taken in a normal clinical setting thus with the X-ray modality (Fuji FCR XG1) used in the everyday clinic at the hospital. However our data are all obtained with the same modality (Fuji) and analyzed by the same software operator, leading us to assume that the differences in measured porosity between subjects reflect a real difference. Another limitation of the DXR method is that this method has not been validated against other more sensitive methods. Thus a validation of the DXR method, against more sensitive methods, is warranted. Further DXR is performed at metacarpal bone and not at the fracture site. This may not be important as DXR-BMD computed from the metacarpals has been found to be strongly correlated with a former version of DXR-BMD computed from metacarpals, radius and ulna (Clinical report Protocol XPO/US-01, manufacturer).

Regarding possible confounders we used in our study variables known to have a possible impact on BMD and thus also might influence porosity, but whether they all actually affect the porosity or other bone quality features is not determined. Adjusting for these variables did not change the association between porosity and fracture.

Uncertainty about dosage and duration of medications that could interfere with bone metabolism in our study may be a problem but the number treated with glucocorticoids or anti-osteoporosis medication was small and did not differ between distal radius fracture group and controls (Table 1).

In summary, our results suggest that increased cortical bone porosity; a measure of bone structure is an important and independent risk factor for distal radius fracture risk in middle aged and elderly women. Further studies are warranted to validate the DXR method and to explore whether DXR-porosity can be clinically useful to identify women at higher risk for distal radius fracture and fractures at other sites, especially in individuals with normal DXA-BMD.
